# Genetic and environmental drivers of migratory behavior in western burrowing owls and implications for conservation and management

**DOI:** 10.1111/eva.13600

**Published:** 2023-11-15

**Authors:** Kelly Barr, Christen M. Bossu, Rachael A. Bay, Eric C. Anderson, Jim Belthoff, Lynne A. Trulio, Debra Chromczak, Colleen L. Wisinski, Thomas B. Smith, Kristen C. Ruegg

**Affiliations:** ^1^ Center for Tropical Research Institute of the Environment and Sustainability, University of California, Los Angeles Los Angeles California USA; ^2^ Department of Ecology and Evolutionary Biology University of California, Los Angeles Los Angeles California USA; ^3^ Department of Biology Colorado State University Fort Collins Colorado USA; ^4^ Department of Evolution and Ecology University of California, Davis Davis California USA; ^5^ Fisheries Ecology Division, Southwest Fisheries Science Center National Marine Fisheries Service Santa Cruz California USA; ^6^ Department of Fish, Wildlife, and Conservation Biology Colorado State University Fort Collins Colorado USA; ^7^ Raptor Research Center and Department of Biological Sciences Boise State University Boise Idaho USA; ^8^ Department of Environmental Studies San José State University San Jose California USA; ^9^ Burrowing Owl Researcher & Consultant Riegelsville Pennsylvania USA; ^10^ San Diego Zoo Wildlife Alliance Escondido California USA

**Keywords:** genetic connectivity, genomics, genotype–environment associations, inbreeding, migration

## Abstract

Migration is driven by a combination of environmental and genetic factors, but many questions remain about those drivers. Potential interactions between genetic and environmental variants associated with different migratory phenotypes are rarely the focus of study. We pair low coverage whole genome resequencing with a de novo genome assembly to examine population structure, inbreeding, and the environmental factors associated with genetic differentiation between migratory and resident breeding phenotypes in a species of conservation concern, the western burrowing owl (*Athene cunicularia hypugaea*). Our analyses reveal a dichotomy in gene flow depending on whether the population is resident or migratory, with the former being genetically structured and the latter exhibiting no signs of structure. Among resident populations, we observed significantly higher genetic differentiation, significant isolation‐by‐distance, and significantly elevated inbreeding. Among migratory breeding groups, on the other hand, we observed lower genetic differentiation, no isolation‐by‐distance, and substantially lower inbreeding. Using genotype–environment association analysis, we find significant evidence for relationships between migratory phenotypes (i.e., migrant versus resident) and environmental variation associated with cold temperatures during the winter and barren, open habitats. In the regions of the genome most differentiated between migrants and residents, we find significant enrichment for genes associated with the metabolism of fats. This may be linked to the increased pressure on migrants to process and store fats more efficiently in preparation for and during migration. Our results provide a significant contribution toward understanding the evolution of migratory behavior and vital insight into ongoing conservation and management efforts for the western burrowing owl.

## 1 INTRODUCTION

Migratory behavior has evolved repeatedly throughout the animal kingdom as species move to maximize their fitness in response to heterogeneous and changing environments (Dingle & Drake, 2007; Pulido, 2007; Shaw, 2016). While evolutionary theory commonly identifies seasonal fluctuations in climate and resources as the primary impetus (Alerstam et al., 2003; Cox, 1985; Winger et al., 2019), much remains to be learned about the relative contributions of environmental, genetic, and associations between genotypic and environmental variation in driving migratory phenotypes. Previous research into these drivers typically focused on the identification of individual environmental or genetic determinants. For example, significant environmental determinants include factors such as changing habitats (Gómez‐Bahamón et al., 2020) and climates in birds (Winger et al., 2019), resource availability (Teitelbaum et al., 2015) and extreme weather events in mammals (Leclerc et al., 2021), and photoperiods and atmospheric pressure in insects (Chapman et al., 2015). Meanwhile, genetic determinants of migratory phenotypes are confirmed through both manipulation experiments, such as captive breeding and crossbreeding studies on both songbirds (Berthold & Pulido, 1994; Berthold & Querner, 1981; Pulido, 2007) and insects (Kent et al., 2001), and, more recently, the identification of numerous candidate genes associated with migratory behavior (Bossu et al., 2022; Jones et al., 2015; Mueller et al., 2011; Toews et al., 2019; Zhan et al., 2014).

Alleles of such candidate genes underlying differing migratory phenotypes may have important associations with environmental variation, but often these are not explicitly examined. In salmon, for instance, variations of the gene *GREB1* dictate the timing of migratory runs to upstream breeding grounds (e.g. spring/summer vs. fall; Narum et al., 2018; Thompson et al., 2020). Atlantic cod (*Gadus morhua*) are either migrants or resident facultatively based upon the orientation of a region of genes associated with increased movement performance, and post‐glacial expansion of migratory populations are thought to be driven by the development of adaptive alleles in these regions that facilitated fitness in Arctic waters (Berg et al., 2017; Kirubakaran et al., 2016). In American Kestrels (*Falco sparverious*), migratory timing is significantly linked to variants in several known genes that regulate biological clocks (Bossu et al., 2022). Although the focus of these studies is strictly on identifying genomic regions associated with migratory phenotypes, the fact that migration occurs in each of these systems as a response to environmental stimuli suggests that genotype–environment interactions may be an important component.

Recent developments in genotype–environment association (GEA) analyses afford a promising opportunity to improve our identification of links between environmental and genotypic variation (Forester et al., 2018). For example, recent work employed GEA analyses to address adaptation‐related questions, including identifying environmental and genetic drivers of adaptation (Capblancq et al., 2018; Dorant et al., 2020), predicting where rapid climate change may cause maladaptation in local populations (Bay et al., 2018; Fitzpatrick et al., 2021; Ruegg et al., 2018; Vanhove et al., 2021), and providing critical information to ensure the success of increasingly necessary and intensive conservation actions such as assisted gene flow (Borrell et al., 2020). Another potential avenue for GEA analyses would be to address hypotheses about the environmental drivers of genetic variation linked to specific phenotypes. We can gain further insight into vital evolutionary phenomena such as variable migratory phenotypes within species by explicitly examining interactions among genotypes, phenotypes, and environmental variation using GEA analyses.

**FIGURE 1:**
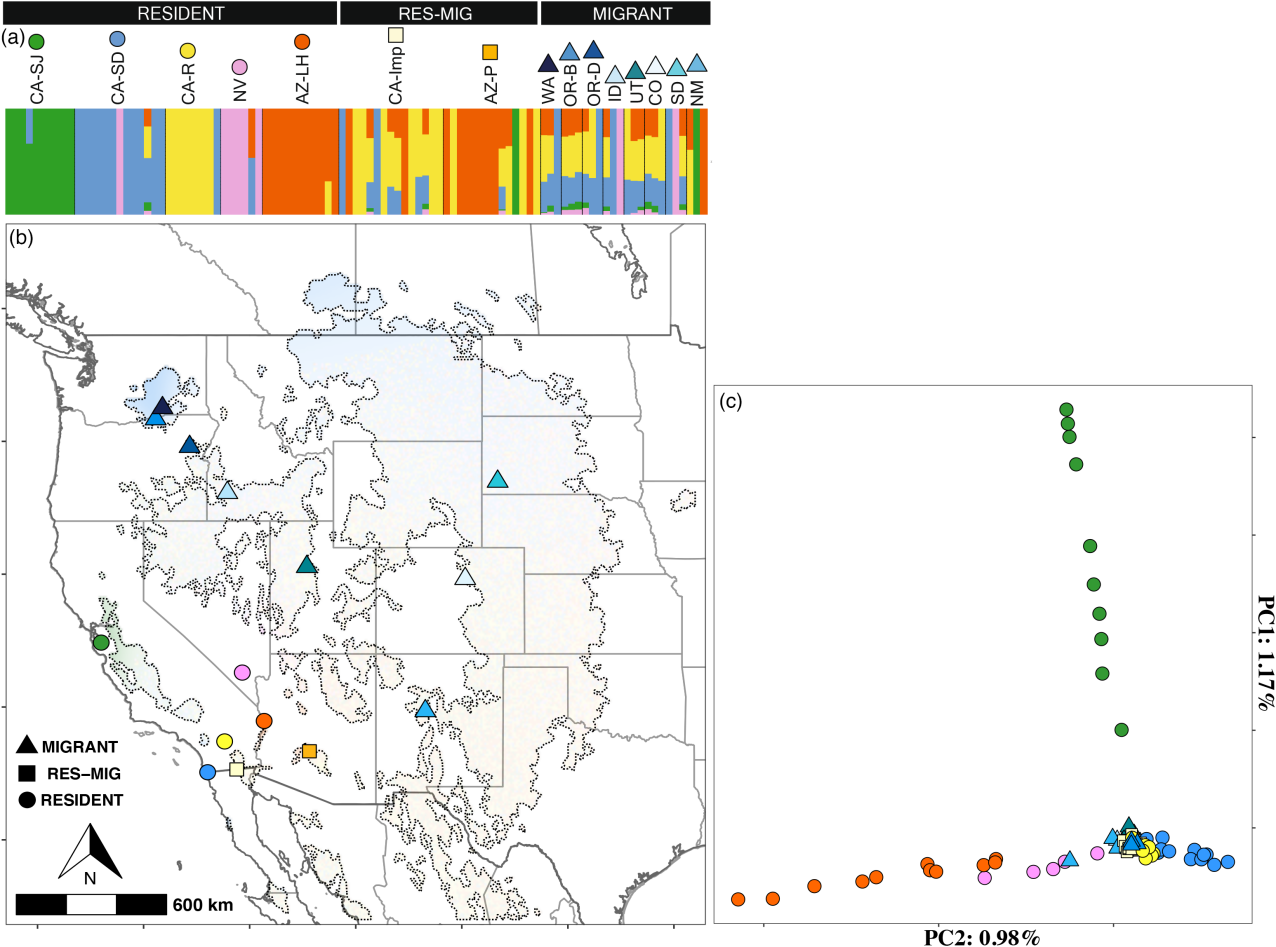
Map of sampling strategy and primary genetic structure results for the western burrowing owl. (a) Plot of NGS‐Admix result at *K* = 5. Migrant sires were reduced to three individuals each to facilitate analyses. Icons next to sample site names correlate with sites on map in (b) and PCA in (c). Samples are arranged by resident, resident sites that were not genetically distinct from the migrant (“RES‐MIG”), and migrant breeding sites. (b) Map of western burrowing owl breeding range (dotted outline) as predicted based upon eBird data. Sample sites are indicated by icons. Shading indicates cluster membership for the five genetically structured resident breeding sites using a kriging of NGS‐Admix results. (c) PCA on all samples across ~3 M variants using single read sampling. Five resident breeding sites are clearly differentiated. The “RES‐MIG” and migratory breeding sites exhibit no differentiation and hence generally overlap at the origin of the PCA.

Here, we analyze links between genotypic and environmental variation underlying migratory phenotypes in the western burrowing owl (*Athene cunicularia hypugaea*), a species designated as being of conservation concern by the U.S. Fish and Wildlife Service (USFWS) and numerous states. Because interpreting GEAs and characterizing the genetic health of populations both require a detailed understanding of gene flow patterns, we also analyze genetic structure and inbreeding to inform our analyses and provide critical information for ongoing species conservation efforts. The western burrowing owl offers an ideal opportunity for this investigation because the subspecies is composed of both resident and migratory phenotypes across an extensive western North American breeding range where it is likely subject to a breadth of ecological variation (Figure 1b).

As many migratory birds, *A. c. hypugaea* exhibits a cline of phenotypes along a latitudinal gradient with largely resident breeders in the southwestern U.S. and fully migratory populations farther north. Migrating burrowing owls are known to make relatively smaller movements versus that of other Neotropical migrants, with an average of 1800 km (334–3541 km; C. Conway personal communications). Resident breeding groups area also frequently exhibit partial non‐breeding migration (Chapman et al., 2011), meaning many individuals remain resident through the breeding cycle and others migrate to breed (Ogonowski & Conway, 2009). Examining genetic relationships among resident and migratory breeding groups is an important goal for conservation and management of the species, particularly given the numerous on‐going captive breeding and translocation projects (Doublet, 2020; Hennessy et al., 2002).

Using a high‐resolution dataset composed of a de novo high coverage reference genome assembly and low coverage whole genome resequencing of samples from numerous migratory and resident breeding populations across the western burrowing owl's range, we address the following questions: (1) How does differential migratory behavior impact gene flow and inbreeding? (2) Are resident and migratory breeding groups genetically isolated from one another? (3) Are there correlations between genotypic and environmental variations that explain differences between migratory phenotypes? Our results not only reveal novel relationships between environmental variation, genotypes, and migratory phenotypes, but also provide critical information for ongoing species conservation efforts by reporting differences in two primary indices for understanding and predicting genetic health: population structure and inbreeding.

## 2 MATERIALS AND METHODS

### 2.1 Variant detection

Details regarding sample collection, genome sequencing, and sequence processing may be found in Methods S1; but notably, we sequenced a reference genome to high coverage and 202 burrowing owl samples collected across their migratory and resident breeding range to low coverage. Because our resequencing dataset was low coverage, we used variant detection and analytical methods that largely did not require called genotypes. This included both genotype likelihoods as estimated in the program ANGSD (Korneliussen et al., 2014) and a single‐read‐sampling (SRS) method that randomly selects one read per variant to temper the bias of high variation in locus‐to‐locus depths. Using these methods, files were prepared for analyses as described below using the following four filtering and genotyping frameworks and conditions: (1) Using ANGSD to produce genotype likelihood files for all individuals in the BEAGLE format (‐doGlf 3) and a minor allele frequency file (‐domaf 1) with restrictive filtering that uses a conservative minimum minor allele frequency (‐minmaf 0.05), a low maximum likelihood of being polymorphic (‐SNP_pval 1e‐6), adjusting mapQ scores for excessive mismatches from the reference genome (‐C 50), and confirming variants using a base alignment quality estimation (‐baq 1). (2) For SRS analyses, we used the ‘HaplotypeCaller’ module in GATK (McKenna et al., 2010) to call genotypes for all individuals sequenced, filtered by removing insert/deletion variants, and kept only biallelic variants found in 50% of the individuals. (3) We used ANGSD to create population‐specific site frequency spectra (SFSs) from site allele frequency files using the reference genome to polarize allele calls (‐anc), adjusting frequencies using individual *F*_IS_ (‐indF), and with strict filtering conditions including discarding reads without unique mapping (‐uniqueOnly 1), removing bad reads (‐remove_bads 1), using only reads for which mates are mapped (‐only_proper_pairs 1), discarding reads with low mapping quality (‐minMapQ 1), keeping reads with high base quality (‐minQ 20), dropping reads with low or high depth across samples (‐setMinDepth 10 ‐setMaxDepth 500), keeping only biallelic sites (‐skipTriallelic 1), and also previously described conditions (‐minMaf 0.05 ‐C 50 ‐baq 1). (4) Minor allele frequency files (MAFs) were also generated for each sample site (‐doMaf 1), sampling all the sites identified in the overall MAF file, and only generating minor allele frequencies for variants found in a minimum of four individuals in each population.

### 2.2 Population structure and inbreeding

We assessed population structure and gene flow patterns using multiple analytical frameworks, including principal components analyses (PCAs), Bayesian clustering analyses, estimation of genetic differentiation (*F*_ST_), and calculating inbreeding (*F*_IS_). Because the inclusion of related individuals can introduce bias in many of the analyses used here, we identified close relatives (i.e., either parent‐offspring or full sibships) using a combination of results from NgsRelate v2 (Hanghøj et al., 2019) and PCAs. A BEAGLE genotype likelihood file was prepared for NgsRelate v2 using the first set of conditions (1) described above for ANGSD. We removed the individual with the lowest coverage from each dyad of high relatedness as indicated by two of the following three analyses: (1) high relatedness across maximum likelihood estimates of the Jacquard's coefficients (*k*1 > 0.4; Jacquard, 2012), (2) a KING‐robust estimator of kinship (*r* > 0.177; Waples et al., 2019), or (3) being paired outliers in PCAs. We calculated relatedness coefficients both across all samples and within sample sites.

For PCAs, a VCF file was prepared using GATK as previously described before implementing SRS. We obtained allele depths statistics (i.e. the “AD” field in the vcf file) for each subset of samples of interest using bcftools (Danecek & McCarthy, 2018), and filtered for sites that are bi‐allelic (‐m 2 ‐M 2), removed rare variants (‐min‐af 0.01) or fixed variants (‐max‐af 0.99), and eliminated sites with high levels of missing data (‐i ‘F_MISSING <0.5’). With these allele depths, we used the R package ‘SRS_Stuff’ (https://github.com/eriqande/srsStuff) to identify population structure using a PCA with the SRS method.

We prepared genotype likelihood files both for estimating individual‐level inbreeding coefficients (*F*_IS_) and for Bayesian clustering analyses using the first (1) set of conditions described above for ANGSD. We estimated *F*_IS_ using ngsF (Vieira et al., 2013) and, based upon our results, compared the means between residents and migrants using a Wilcoxon sign test in the R package ‘ggpubr.’ For clustering analyses, we ran 10 repetitions of NGSadmix (Skotte et al., 2013) each number of clusters (*K*) from 2 to 8 and compared these visually using the R package ‘pophelper’ (Francis, 2017) to assess consistency across multiple runs. Based upon our results (i.e., no structure among migrants; see Section 3), we reduced migratory breeding sites to three individuals each to make analyses more tractable. Note this reduction was only for the NGSadmix analysis and doing so would not be expected to impact results. Once we determined the most consistent K across runs, we mapped the results to create a GENOSCAPE (Ruegg et al., 2014, 2021). For this, we created a novel breeding range map for *hypugaea* using the R package ‘ebirdst’ (Fink et al., 2020) that utilizes citizen science observation data made available through the popular medium eBird (www.ebird.org), and smoothed this map by removing holes using the R package ‘nngeo’ (Dorman, 2018) and small polygons (<400 km^2^) using the R package ‘smoothr’ (Strimas‐Mackey, 2021). Then we used a modification of the R package ‘tess3r’ (Caye et al., 2016) as implemented in ‘TESS3_encho_sen’ (github.com/eriqande/TESS3_encho_sen) to map the cluster membership identified in NGSadmix using spatial kriging.

Finally, we calculated pairwise *F*_ST_ between all sample sites and tested for isolation by distance (IBD) among sites. For this, we estimated SFSs for each sample site as previously described (3) and then we used the ‘realSFS’ suite in ANGSD to create two dimensional SFSs and estimate *F*_ST_s for each pair of sample sites. Using a Mantel test in the R package ‘vegan’, we assessed the significance of IBD across all sites and among either migratory or resident breeding sites only.

### 2.3 Identifying candidate loci

We used an available annotated genome for another burrowing owl subspecies, *A. c. cunicularia* (Mueller et al., 2020), to determine if loci that are highly differentiated between migrants and residents are in genic regions. We used this genome and the annotation because it is of higher quality than we sequenced for *A. c. hypugaea* (see Section 3). For this analysis, we used the top 99.9% *F*_ST_ loci in a comparison of resident and migratory breeding groups. Using two pools of samples, one composed of the five resident populations that exhibit multiple lines of evidence for genetic structure and the other composed of all migratory sites, we created two‐dimensional SFSs using ANGSD as previously described (genotyping conditions set 3), and calculated *F*_ST_ locus‐by‐locus using realSFS. Two sites were excluded from the “resident” pool because they were not genetically distinct from the migratory breeding sites in multiple analyses, and we were focused on detecting the genetic variants that differentiate migratory versus resident breeding groups. We then used BEDTools (Quinlan & Hall, 2010) to clip 200 bp segments around each locus and mapped them to the *A. c. cunicularia* genome, which was downloaded from genbank, using the ‘aln’ module in bwa because this outperforms ‘mem’ for such short segments. Using BEDTools, we then collected a candidate list of genes from the *A. c. cunicularia* annotation found within 25,000 bp segments around the mapped 200 bp segments. We compared this list of candidate genes to a growing list of genes known to be associated with migratory behavior (following Bossu et al., 2022). Finally, we used ShinyGO (Ge et al., 2020) to perform a gene ontology analysis using both the chicken (*Gallus gallus*) and zebra finch (*Taeniopygia guttatus*) gene sets for comparisons.

### 2.4 Genotype–environment associations with migratory behavior

For GEAs of migratory phenotypes, we used gradient forest analyses to detect correlations between environmental variables and outlier loci in a comparison of migratory and resident genotypes. We defined outlier loci for this analysis as the top 1% most differentiated loci in the locus‐by‐locus *F*_ST_ calculation previously described between pools of migrants and residents (again, excluding unstructured resident sites based upon our results). Since low coverage whole genome data invariably include many missing sites per individual, we used population‐specific allele frequencies for each sample site for these analyses. Outlier loci were subset from the sample‐site specific MAF; however, since MAF files are calculated based upon the individual sample sites and filtering conditions vary by numbers of samples at each, not all outlier loci identified in the full migrant‐resident comparison were present for individual sites. Those missing were dropped from further analysis. Numerous loci and one migratory site (NM) were removed from further analysis because of high levels of missing data (>50%). Remaining missing allele frequencies were imputed using the R package ‘mice’ (van Buuren & Groothuis‐Oudshoorn, 2011) using default settings.

We used the R packages ‘gradientforest’ and ‘extendedforest’ (Ellis et al., 2012) to test for correlations between these outlier loci and each of 19 bioclimatic variables (Hijmans et al., 2005), elevation, Normalized Difference Vegetation Index (NDVI), tree cover, migratory status (resident or migatory), and 20 landcover categories captured in the National Land Cover Database (NLCD; Dewitz, 2021). Details about these environmental variables can be found in Supplemental Material. Landcover was estimated for each category within 20 km radii around the center points of sample sites to include most of the area being used by burrowing owls. We also included the first two PCs from PCAs using all sample sites to account for genetic structure, and, to account for spatial biases, included the first two PCs from a principal coordinate of neighborhood matrix (PCNM) conducted using ‘vegan.’ For gradient forest analyses, we collected 2000 trees (nbin = 1, corr.threshold = 0.5) for each genetic variant and obtained a ranked list of environmental variables based upon their relative predictive power. This analysis was run 10 times to assess consistency in the top environmental variables identified. Using 10 replicates that each randomly permutes the observed environmental variation among sites, we assessed the significance of correlations by comparing the number of SNPs with a non‐zero *R* ^2^ and the mean *R* ^2^ across these loci. For visualization of these results, the top four environmental variables were then collected from 10,000 random points from within the breeding range we predicted based upon observation data as previously described. This is bound to range of *A. c. hypugaea* within the United States due to the limitations of the NLCD, which only included information for the contiguous 48 states.

## 3 RESULTS

### 3.1 Data quality

We produced an *A. c. hypugaea* reference genome assembly with an average depth of 49×. The total length of 1.25 Gb is spread across 3830 scaffolds at an N50 of 2.6 Mb. BUSCO analyses revealed that 96.8% of the known genes from class Aves are captured by this reference genome. For the resequencing data, we removed low quality libraries (*N* = 18) and one member of related pairs identified (*N* = 23) and then conducted the following analyses with a dataset of 161 individuals sequenced to an average depth of 0.98× (range: 0.0104×–2.132×).

### 3.2 Genetic structure and diversity

**FIGURE 2:**
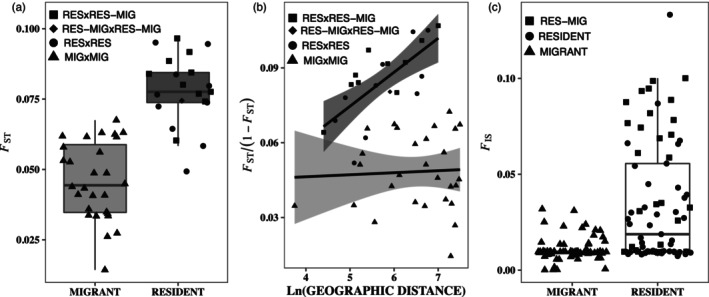
Comparisons of *F* statistics between BUOW migratory and resident breeding sites. Exception resident sites that are not structured from the migrant sites are grouped with resident breeding sites. (a) Residents are significantly more differentiated from one another than migrants (*W* = 26, *p* < 0.001). (b) Residents exhibit significant isolation‐by‐distance (*r* = 0.67, *p* = 0.004) while migrants do not (*r* = −0.04, *p* = 0.58). (c) Inbreeding is significantly higher in residents than migrants (*W* = 1285, *p* < 0.001).

Population structure was strongly associated with migratory behavior. NGSadmix (Figure 1a,b and Figure S1; based upon 1,315,863 SNPs), the PCA (Figure 1c; based upon 3,473,488 SNPs), and pairwise *F*_ST_s (Figure 2a,b and Table S2; based upon an average of 535,754 SNPs), all identified limited gene flow among resident breeding sites and no indications of limitations among migratory breeding sites. Other than two exceptions, the resident breeding sites are easily discriminated from one another and from the migratory sites when comparing PCs (Figure 1c), exhibit higher relative levels of genetic differentiation (Figure 2a), and form distinct clusters when considering 2–8 Ks (Figure S1). Conversely, the migratory sites are not distinguishable from one another in a PCA (Figure 1c) exhibit lower relative genetic differentiation (Figure 2a), and exhibit high levels of admixture at all Ks considered (Figure S1). We note that the NGS‐Admix results are expectedly messy given the low coverage data being analyzed; but the overall trends in population structure are clear and supported by additional analyses.

These same patterns are further supported by analyses of IBD and inbreeding. While migratory sites do not follow a pattern of IBD, the correlation between genetic differentiation and geographic distance is significant and positive among the resident breeding sites (Figure 2b; Mantel's *r* = 0.675, *p* < 0.01). This suggests high gene flow in the former and distance‐restricted/stepping‐stone gene flow in the latter. Meanwhile, restrictions on gene flow in residents are also evidenced by higher measures of individual inbreeding coefficients, *F*_IS_, in resident versus the migrant populations (Figure 2c; *W* = 1285, *p* < 0.001).

Two sites, one composed of samples collected from burrowing owls around Phoenix, AZ (AZ‐P) and another in the Imperial Valley, CA (CA‐Imp), are exceptions among the resident breeding sites. These areas cannot be distinguished from the migrants using PCA (Figure 1c) and do not form individual clusters in NGSadmix (Figure 1a and Figure S1); however, these sites exhibit higher levels of inbreeding than observed in migratory sites, especially at AZ‐P where inbreeding appears to be the highest of all study sites (Figure S2). Genetic differentiation (*F*_ST_) is also high between these two areas and the migratory sites (Table S2).

### 3.3 Phenotype–genotype–environment analyses

**FIGURE 3:**
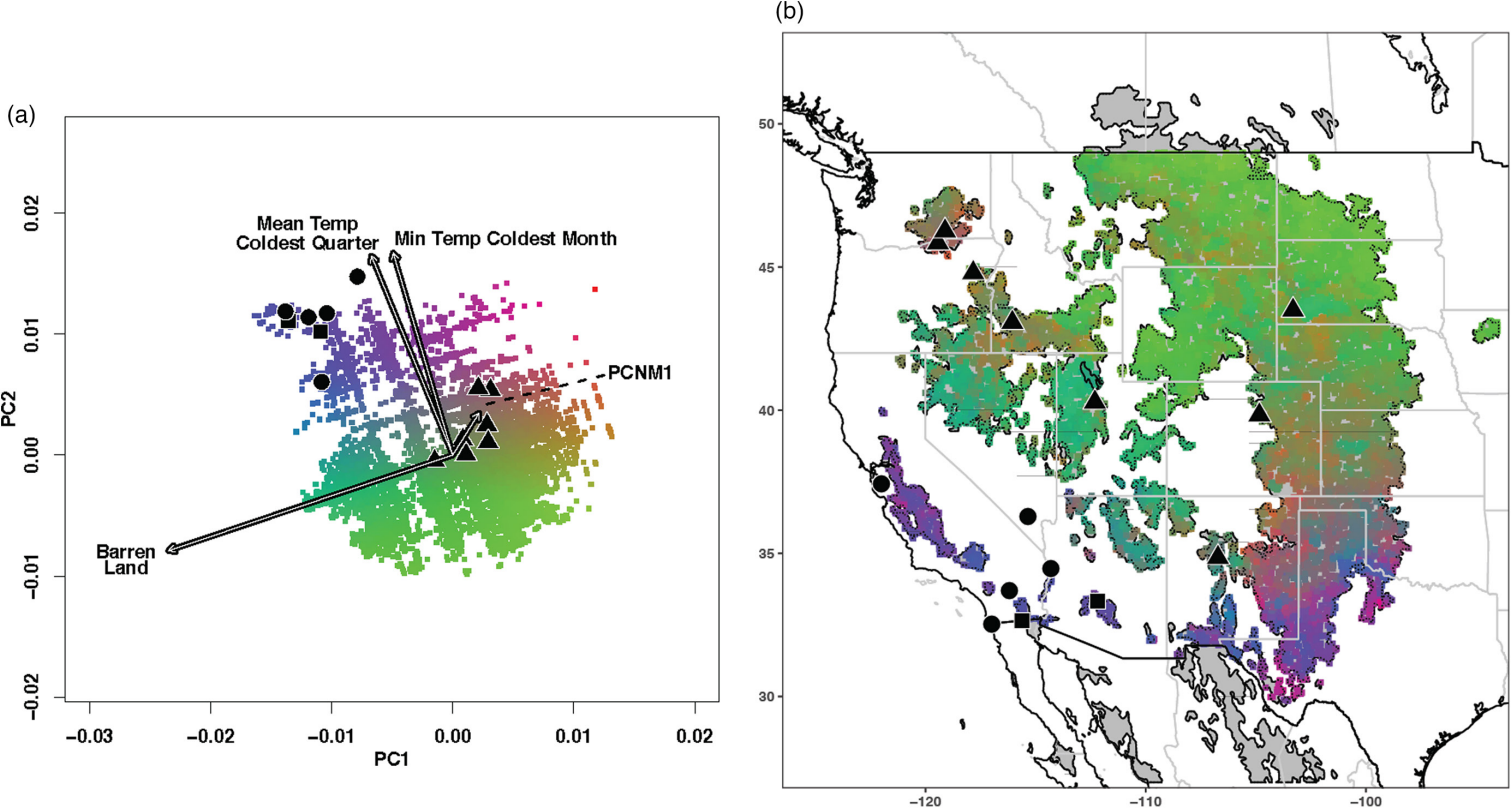
PCA (a) and map (b) portraying gene–environment correlations associated with migratory behavior across the BUOW range. Colors are based upon 10,000 random points across the breeding range, but is restricted to the U.S. due to the availability of the landcover data. (a) PCA of climate variables with PC scores associated with sample sites indicated with symbols that match Figure 1. Arrows indicate the loadings of top‐ranked variables identified by gradient forest analysis. (b) Map of projected GEA correlations across the BUOW range and sample sites indicated as in Figure 1.

After subsetting population‐specific minor allele frequency files generated in ANGSD for the top 1% highest *F*_ST_ loci between residents and migrants and dropping loci related to missing data, we used 6954 SNPs for gradient forest analyses. These were the highest of 815,438 total loci with positive *F*_ST_s (mean *F*_ST_ = 0.024) considered for this analysis. Top loci were distributed across the genome and had an average *F*_ST_ of 0.205 (SD = 0.039). Of these, 3458 were positively correlated with environmental variables. Repeated gradient forest runs (*N* = 10) consistently identified the same top 10 environmental variables in terms of *R* ^2^ weighted importance in the same order between runs (Figure S3). The top four uncorrelated variables were, in order of ranked importance, minimum temperature of the coldest month (Bioclim 6), pcnm1 (independent spatial variable pc1), barren/open land (landcover class 31), and mean temperature of the coldest quarter (Bioclim 11). The relative strength of these variables for explaining genetic differences between the migratory and resident breeding sites is visualized with the PCA in Fig. 3a, and these are projected across the burrowing owl's breeding range to further examine the relationships with migratory phenotypes (Fig. 3b). These were the top four variables in order across all 10 empirical gradient forest runs, and comparisons of these variables between migratory and resident breeding sites illustrate apparent differences at each one of these (Figure S6a–d). Note that PC1, which accounts for population structure, is the 11th most important explanatory variable, and 9 of 10 variables that are higher are environmental (Figure S3). Empirical observations of SNPs with positive *R* ^2^ and the average *R* ^2^ across loci were significantly higher than in randomizations (Figure S4). It is not surprising that pcnm1, which accounts for spatial autocorrelation among the sample sites, would be a top variable as we examined breeding aggregations with a clear spatial relationship (i.e., migratory populations in higher latitudes and resident populations in lower). Including pcnm1 in our analyses is a conservative approach, as genetic variants that might otherwise be associated with other environmental predictors are linked to it instead. Furthermore, the top four most important loci for each of the top four environmental variables identified by gradient forest analyses exhibit allele frequency differences between residents and migrants that trend with environmental variation (Figure S5).

### 3.4 Candidate loci

The *A. c. cunicularia* genome, with an N50 of 42M bp over 445 scaffolds, is significantly less fragmented than the one we produced for *A. c. hypugaea* (see Section 3). Hence, using the higher quality genome of this subspecies is well‐justified. Of the 0.1% most differentiated loci between migrants and residents (*N* = 1009), 960 mapped successfully to the *A. c. cunicularia* genome. Within 25,000 bp regions around these successfully mapped sites, 457 unique genic regions were identified using the *cunicularia* annotation, and 116 of these had unique NCBI identifiers. We obtained a list of 24 recognized genes within these genic regions (Table S3). Gene ontology analyses using the chicken and zebra finch genomes both revealed enrichment in several pathways associated with processing fat (i.e., lipophagy; Table S4). This was the only pathway with significant enrichment. There were no genes in common with a list of previously identified genes relevant to migratory behavior in birds following Bossu et al. (2022).

## 4 DISCUSSION

While it is generally accepted that migratory behavior is an adaptation to life in seasonal environments, few studies have successfully identified genetic and environmental associations underlying this key fitness‐linked trait. Here we combined population and landscape genomic approaches to identify putative environmental associations with genetic differentiation between migrant and resident burrowing owls across North America. We found strong associations between the top differentiated loci between migrant and resident breeding burrowing owls and environmental variables related to cold, winter temperatures (i.e., the coldest month and the coldest quarter; Figure S6a,d) and barren, open habitat (Figure S6c). Furthermore, gene flow, population structure, and inbreeding patterns largely could be explained by breeding strategy. Migratory breeders exhibited high gene flow and low inbreeding, and resident breeders exhibited limited gene flow and high inbreeding. Further investigation into the putative function of genes underlying migratory behavior provides further insight into differences in the forms, specifically indicating differences in genes linked to metabolic processes (i.e., liphophagy). Overall, our results have important implications for understanding links between genetic and environmental variation underlying migratory behavior across species and for the genetic health (i.e., inbreeding and gene flow) of western burrowing owl populations.

### 4.1 Population structure, gene flow, and inbreeding

While previous genetic studies failed to identify any significant limitations to gene flow among resident or migratory breeding groups in western burrowing owls (Desmond et al., 2001; Korfanta et al., 2005; Macías‐Duarte et al., 2020), we detected clear population structure patterns associated with migratory phenotypes. Namely, we found distinct genetic clustering of residents by population and no limitations to gene flow among the migratory breeding groups. Many organisms exhibit similar gene flow regime differences based upon migratory behavior with genetic structure among resident breeders and high connectivity among migrants, including brown trout (*Salmo trutto*; Lemopoulos et al., 2018), river lampreys (*Lampetra fluviatilis*; Bracken et al., 2015), European blackcaps (*Sylvia atricapilla*; Delmore et al., 2020), and numerous bat species (Moussy et al., 2013). This is also not unexpected for western burrowing owls given the propensity of individuals from migratory breeding groups to disperse to new areas, sometimes over great distances (Riding & Belthoff, 2018). On the other hand, significant IBD among residents suggests a stepping‐stone pattern of gene flow that leaves distantly‐spaced breeding areas more differentiated from one another. Due to limitations with our low coverage dataset, we did not assess whether resident populations are differentiated due to genetic drift and time or natural selection.

The differences in gene flow between residents and migrants have important implications for the relative genetic health of western burrowing owl populations. Notably, inbreeding is significantly higher in all resident populations than in what seems to be effectively one large migratory population. The level at which inbreeding might have fitness consequences for local populations is difficult to discern and likely varies species‐to‐species. Ralls et al. (2018), however, suggest that an inbreeding level of 0.1 is the point at which an isolated population should receive require an active management response, such as genetic rescue (Whiteley et al., 2015), to avoid decreased fitness. The highest *F*_IS_ we observed was a resident breeder near San Jose, CA at 0.11, and many resident breeding birds were just below this estimate. For species management purposes, it would be valuable to assess if elevated inbreeding has fitness consequences for *A. c*. *hypugaea*, such as lowered breeding success as observed in red deer (*Cervus elaphus*; Slate et al., 2000) or reduced survival as reported in song sparrows (*Melospiza melodia*; Keller, 1998). Furthermore, captive breeding, genetic rescue, and translocation projects, the latter of which sometimes entails novel pairing of adults (Hennessy et al., 2002), would benefit from using genomic data to guide pairing decisions and precisely reduce inbreeding in potential offspring (Bossu et al., 2023).

### 4.2 Genotype–environment drivers of migratory behavior

Although previous studies focused individually on environmental or genetic variants underlying migratory behavior, we identified significant evidence for links between genotypic and environmental variation that differentiate migratory phenotypes within a species. Among the top four environmental predictors (Figure S3) of genetic variation underlying migratory behavior are minimum temperature of the coldest month and mean temperature of the coldest quarter. These top climatic predictors may reflect extreme winter conditions associated with seasonality on migratory breeding grounds, which, in turn, results in the annual fluctuation of resources that is a primary driver of the evolution of migration in birds (Alerstam et al., 2003; Shaw, 2016; Winger et al., 2014, 2019). While the migratory birds are not directly experiencing selection from these environmental variables per se, examining correlations between this and genetic variation helps to further understand the differences between migratory phenotypes. The robustness of this result is illustrated by the randomizations we employed in the gradient forest analyses, as we observed significantly weaker associations and fewer variants with positive associations than those of the empirical dataset.

Another top environmental predictor of genotypic variation was barren land, which is defined as having less than 15% vegetation cover and may reflect low productivity of the desert or otherwise arid landscapes in which many of the resident populations are found. Migratory breeding groups are generally found in more productive grassland habitat—though it is notable that many areas in the migratory breeding range are subject to periods of drought that can also leave the landscape visibly barren as well. It is possible that the connection to barren land is also linked to seasonality. After all, as do all Neotropical migrants during Spring, western burrowing owls migrate northward to take advantage of seasonal abundance. Burrowing owls that are migratory depart the open, barren habitat common to the American southwest to breed in more productive grasslands farther north.

The top outlier loci in our analysis were found to be associated with the regulation of fats, which suggests that migrants and residents may differ in metabolic processes linked to fat mobilization. Specifically, genic regions near the 960 outlier loci in our analysis were enriched for genes involved in the lipophagy pathway (Table S4), which regulates the presence of fat molecules in the body whether via accumulation or metabolism. This result aligns well with previous research into the physiological adaptations of migratory species. Not only are they uniquely able to cache fats for a ready energy source for migration but migrants also more efficiently process them during extended movements (Guglielmo, 2018; Ramenofsky, 1990). A transcriptome study using livers from a passerine collected before, during, and after migration found that the lipophagy pathway specifically was active throughout (Frias‐Soler et al., 2022). Given these observations in other species, one potential explanation for our result is that migratory and resident breeding western burrowing owls use their lipophagy pathways in different ways in relation to adaptation to their contrasting life cycles. Notably, most of the resident breeding sites represented in this study are occupied by migratory birds during the winter, lending further credence to the suggestion that the migratory breeders have experienced enrichment of the lipophagy pathway versus the residents.

Future genomic work on burrowing owls would benefit from a novel genome using long‐read data that would better capture repeat or otherwise hard to sequence regions of the genome that are likely not represented in the reference genome assembled here from short‐read data. Pairing such a reference genome with higher depth data from individuals would be helpful for further understanding the potential fitness effects of elevated inbreeding at many of the resident sites and for further revealing associations between environmental and genetic variant underlying migratory phenotypes.

### 4.3 Lack of structure in two resident sites

Two sample sites, the Imperial Valley of CA (CA‐Imp) and Phoenix, AZ (AZ‐P), are exceptional in being resident breeding sites that cannot be distinguished from the migrants in either admixture analyses (Figure 1a) or PCA (Figure 1c). Arid regions subject to intense irrigation particularly for agriculture are known to support thriving populations of western burrowing owls (DeSante et al., 2004; Macias‐Duarte, 2011). The Imperial Valley, for instance, experienced a 2.5× fold increase in burrowing owl population density from 1980 to 2000 as agricultural operations escalated in the area (Rosenberg & Haley, 2004), and it currently supports the majority of the total extant population in California (Poulin et al., 2020). Recent work suggests non‐breeding partial migratory populations may be experiencing a switch to breeding partial migratory populations in desert areas heavily impacted by agriculture (Macías‐Duarte et al., 2020). Increased gene flow resulting from this change may explain the lack of differentiation in the PCA between both CA‐Imp and AZ‐P and the migratory group. The recency of this phenomenon might be indicated by the fact that these two sites exhibit the same pattern of IBD as other resident breeding sites as it is possible that the sites have yet to reach equilibrium, and there would be a longer lag effect in the *F*_ST_ calculation versus PCA. We cannot resolve the cause from our current dataset, however, and there are other complicating factors. For example, AZ‐P has long been subject to an on‐going, intense translocation project without any guidance on population structure, genetic relatedness, or verification of migratory phenotypes (Doublet, 2020). Notably, AZ‐P exhibits the highest levels of inbreeding for any of the sites that may be the product of inadvertent mixing of close relatives.

### 4.4 Conservation implications

The results we report here have broad implications for our understanding of the evolution of migration and the management of western burrowing owls. Resident breeding populations show elevated inbreeding and may benefit from genetic rescue efforts. Based upon the low genetic differentiation among populations (Frankham et al., 2011), it is unlikely that translocations between structured populations would lead to outbreeding depression; however, our dataset is not sufficient for detecting signals of local adaptation that may exist within resident breeding groups. Future work on potential local adaptation in resident populations could be helpful for guiding source choices for genetic rescue. At many of the sites, genomic data might be used for distinguishing residents from overwintering migrants, which is a considerable difficulty for burrowing owl conservation programs. Given the significant GEAs underlying migratory behavior we detect, future work toward understanding the fitness consequences of retaining migrants to boost nonbreeding partial migratory populations would be a helpful next step for species conservation efforts as well. Further examination of these associations particularly in areas where burrowing owl migratory behavior may be shifting would be beneficial for understanding the links with changing climate and habitat, and also for predicting potential behavioral changes in the species.

## 5 CONCLUSIONS

Our study combines landscape and population genomic approaches to identify associations among genetic and environmental factors underlying migratory phenotypes. Additionally, our GO term analysis suggests enrichment of genes in the lipophagy pathway, lending further support to the idea that migrants and residents differ in their ability to process and store fats. Future work employing similar population and landscape genomic analyses across taxa will reveal the extent to which our findings are generalizable across species.

## ANIMAL WELFARE AND PERMIT STATEMENTS

Samples were collected under Tom Smith's Federal Bird Banding Permit, #21901, and Kelly Barr's Scientific Collecting Permit, #SC‐11568. Animal handling and sampling protocols were conducted with the approval of UCLA's Animal Research Committee (ARC), agreement #2017‐073‐03.

## Supporting information


Data S1.

## Data Availability

All of the genetic data collected for this study are available in public databases. These include raw data sequence files in the NCBI Sequence Read Archive, accession PRJNA1039951, and both genotype (ie, vcf, BEAGLE, site frequence spectra, and allele frequency files) and environmental data files on Dryad, https://doi.org/10.5068/D19X1J.
