# Supplementary material for: Genetic and environmental drivers of migratory behavior in western burrowing owls and implications for conservation and management
Source: Evol Appl. 2023 Nov 15;16(12):1889–900. doi: 10.1111/eva.13600 (PMC10739168; doi:10.1111/eva.13600)
Supplement: Supplementary file 1 — Data S1. [file EVA-16-1889-s001.zip › BUOW-figs-revision.pdf]

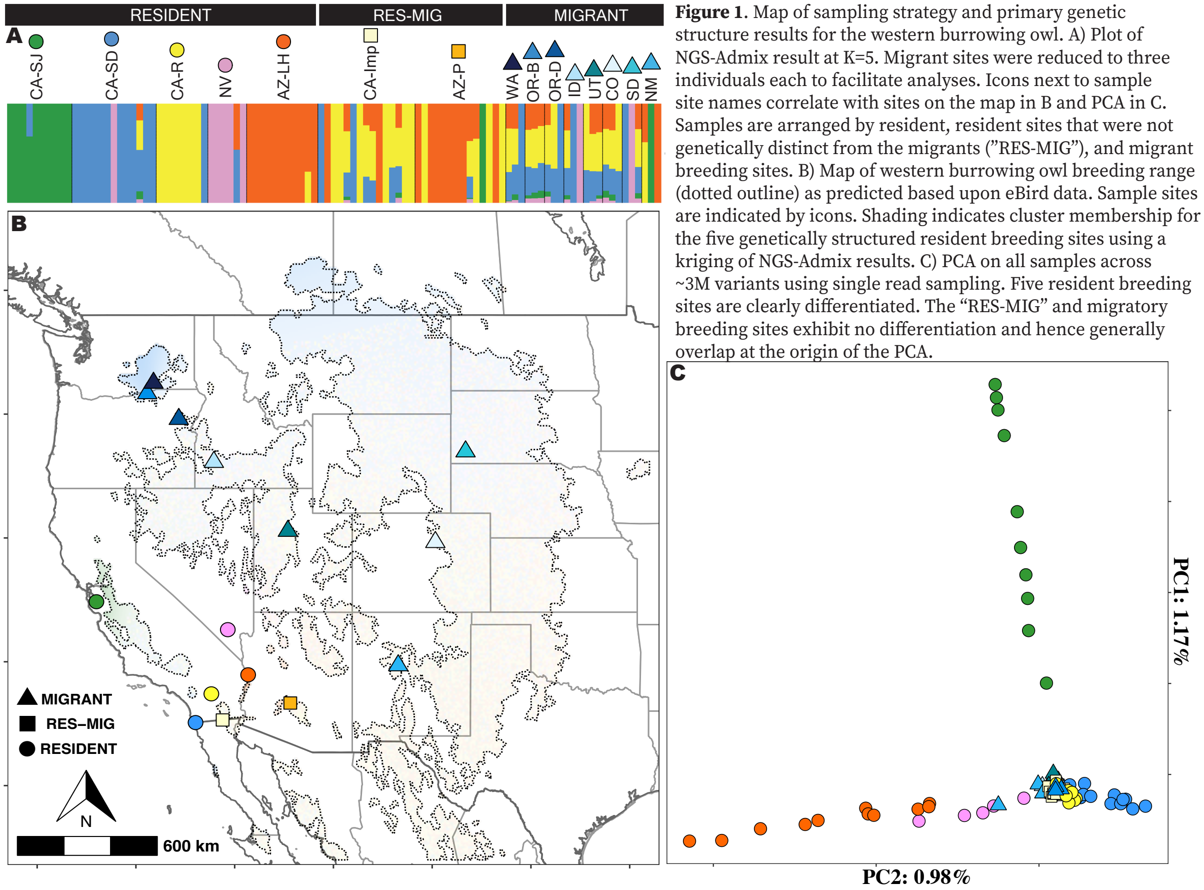

Figure 1. Map of sampling strategy and primary genetic structure results for the western burrowing owl. A) Plot of NGS-Admix result at K=5. Migrant sites were reduced to three individuals each to facilitate analyses. Icons next to sample site names correlate with sites on the map in B and PCA in C. Samples are arranged by resident, resident sites that were not genetically distinct from the migrants ("RES-MIG"), and migrant breeding sites. B) Map of western burrowing owl breeding range (dotted outline) as predicted based upon eBird data. Sample sites are indicated by icons. Shading indicates cluster membership for the five genetically structured resident breeding sites using a kriging of NGS-Admix results. C) PCA on all samples across ~3M variants using single read sampling. Five resident breeding sites are clearly differentiated. The "RES-MIG" and migratory breeding sites exhibit no differentiation and hence generally overlap at the origin of the PCA.

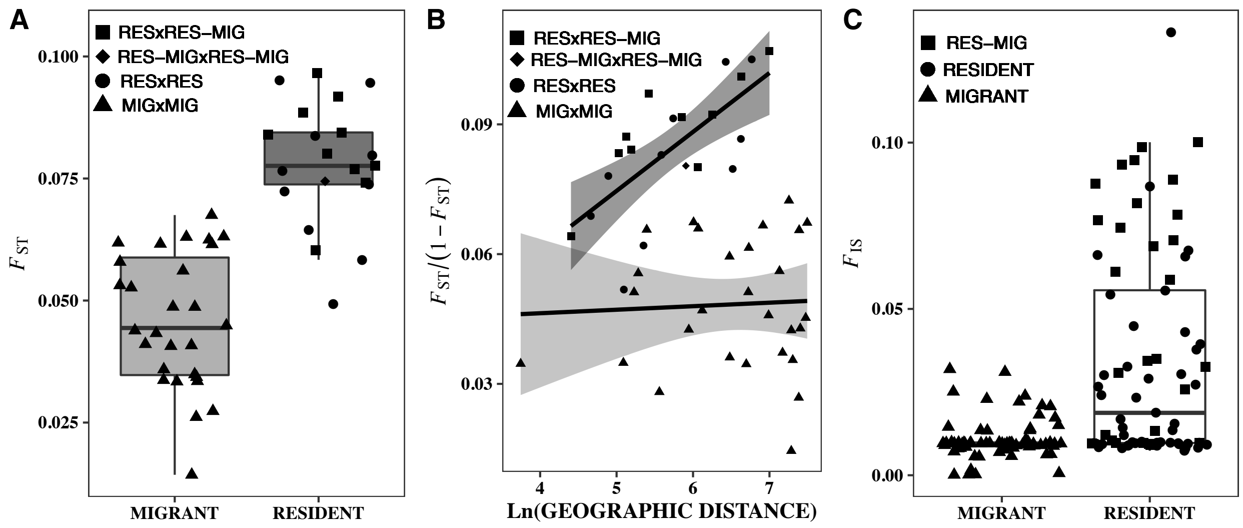

Figure 2. Comparisons of F statistics between BUOW migratory and resident breeding sites. Switcher sites are grouped with resident breeding sites. A) Residents are significantly more differentiated from one another than migrants (W=26, p<0.001). B) Residents exhibit significant isolation-by-distance (r=0.67, p=0.004) while migrants do not (r=-0.04, p=0.58). C) Inbreeding is significantly higher in residents than migrants (W=1285, p<0.001).

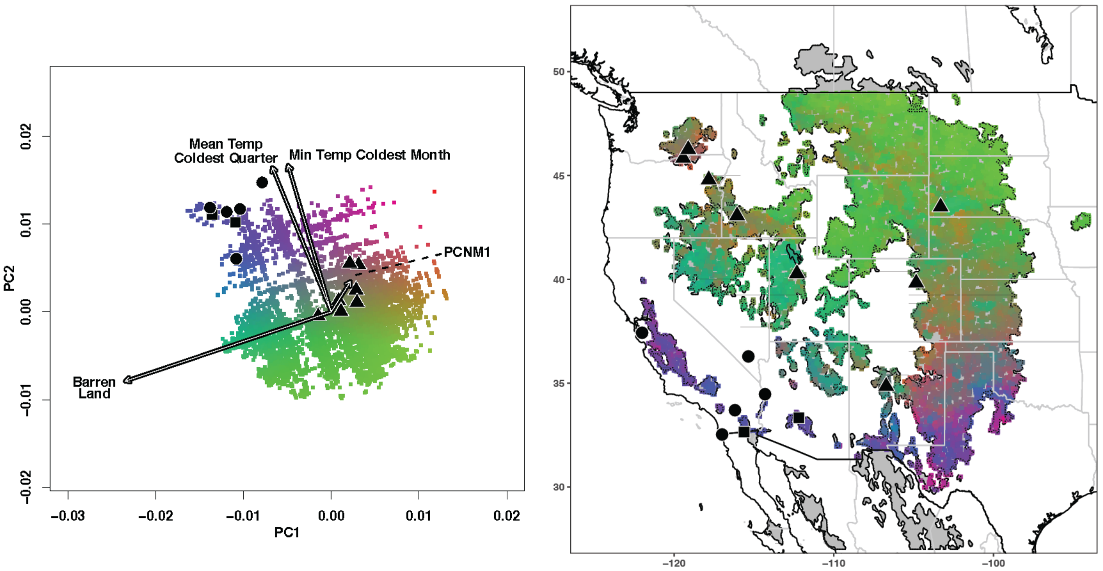

Figure 3. PCA (A) and map (B) portraying gene-environment correlations associated with migratory behavior across the BUOW range. Colors are based upon 10,000 random points across the breeding range, but is restricted to the U.S. due to the availability of the landcover data. A) PCA of climate variables with PC scores associated with sample sites indicated with symbols that match Fig. 1. Arrows indicate the loadings of top-ranked variables identified by gradient forest analysis. B) Map of projected GEA correlations across the BUOW range and sample sites indicated as in Fig. 1.

Table S1. Collaborators who either provided samples or provided access to sites for sampling (“collected with assistance”). Some collaborators provided samples that are not included in this study. *Specific location is the centerpoint GPS for the general sample sites, and these were used for genotype-environmental analyses.

Organization	Contact(s)	Samples Provided	Samples Collected With Assistance	Samples in Analyses	Location(s)	Specific Location*
San Diego Zoo Institute for Conservation Research	Colleen Wisinski	70	0	14	CA-SD	32.55, -116.98
				14	CA-Imp	32.65, -115.61
				8	CA-R	33.71, -116.18
San Jose State University	Dr. Lynn Trulio<br>Debra Chromczak	51	0	10	CA-SJ	37.429, -121.998
Biose State	Dr. Jim Beltoff	147	0	12	ID	43.065, -116.054
				9	ORB	44.8, -117.83
				9	ORD	45.84, -119.43
				7	WA	46.26, -119.11
				12	SD	43.49, -103.31
				10	CO	39.83, -104.84
UCLA/Wild-at-Heart	Bob Fox, Beth Edwards	0	21	14	AZ-P	33.333, -112.183
UCLA/University of Idaho	Carl Lundblad	2	37	10	UT	40.281, -112.306
				6	LV	36.301, -115.346
				6	NM	34.852, -106.719
UCLA/Arizona State University at Lake Havasu	Kerrie Anne Loyd	2	12	11	AZ-LH	34.479, -114.317
Assiniboine Zoo	Stephen Petersen	18	0	0	Assiniboine Zoo	Winnipeg, MB, Canada
University of Florida	Elizabeth White	6	0	0	Florida	Florida, USA
Calgary Zoo	Graham Dixon-MacCallum	18	0	0	Calgary Zoo	Calgary, AB, Canada
The Burrowing Owl Conservation Society of BC	Lauren Meads	11	0	0	British Columbia/<br>Oregon	British Columbia, Canada<br>Umatilla, OR, USA

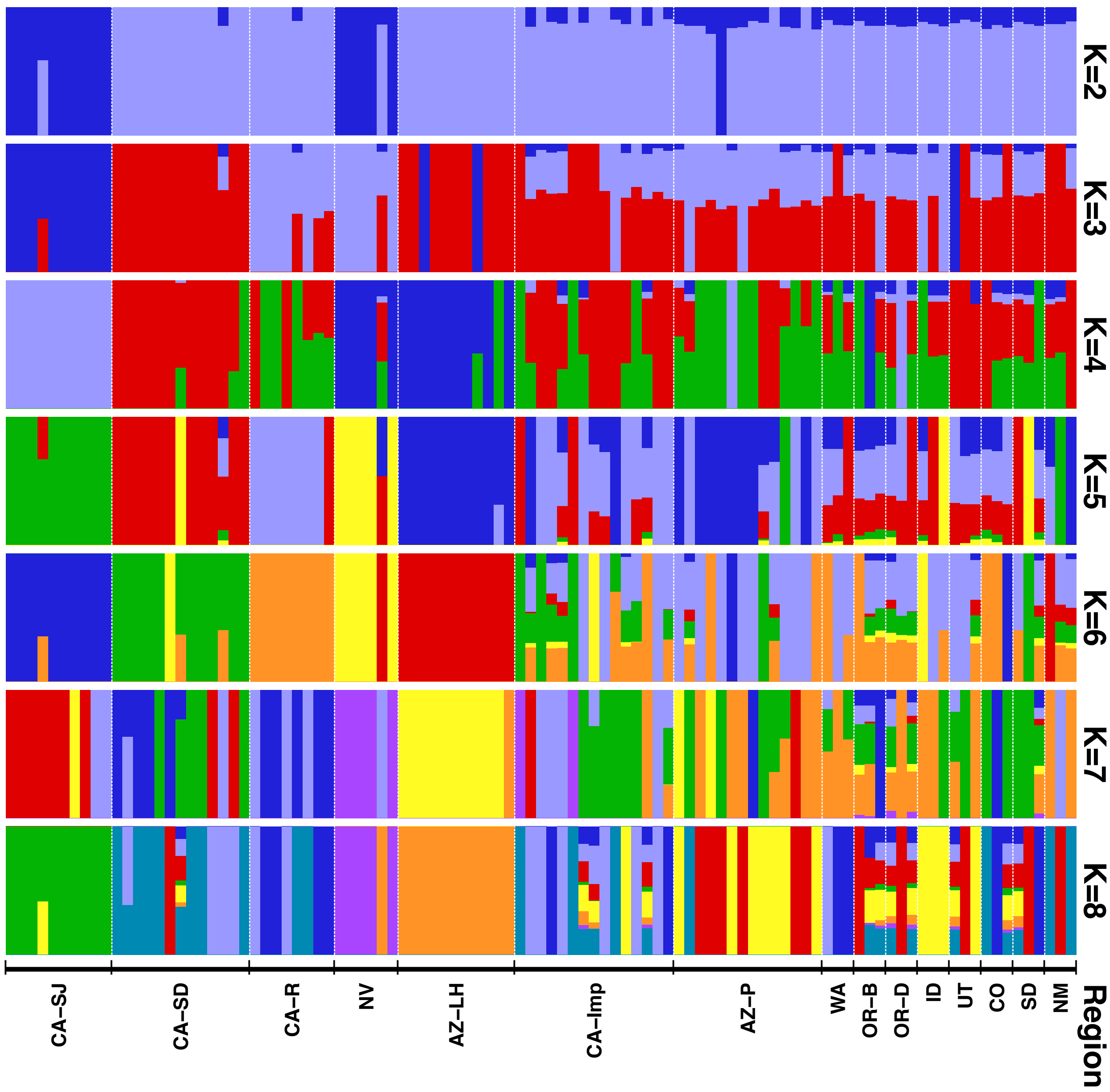

Figure S1. NGS-Admix results at Ks between 2 - 8, as indicated on the right. Migratory sites were reduced to 3 samples each to facilitate analyses.

Table S2. Pairwise F_{ST} between all sites sampled as calculated using the 'realSFS' module in ANGSD.

	CO	ID	CA-Imp	AZ-LH	NV	NM	CA-SJ	OR-B	OR-D	AZ-P	CA-R	SD	UT	WA
**ID**	0.0530													
**CA-Imp**	0.0551	0.0684												
**AZ-LH**	0.0587	0.0776	0.0801											
**NV**	0.0455	0.0730	0.0776	0.0732										
**NM**	0.0274	0.0439	0.0482	0.0441	0.0332									
**CA-SJ**	0.0661	0.0902	0.0923	0.0951	0.0797	0.0647								
**OR-B**	0.0407	0.0616	0.0651	0.0658	0.0515	0.0359	0.0756							
**OR-D**	0.0404	0.0642	0.0667	0.0681	0.0527	0.0343	0.0748	0.0488						
**AZ-P**	0.0590	0.0760	0.0745	0.0885	0.0839	0.0667	0.0961	0.0706	0.0735					
**CA-R**	0.0383	0.0597	0.0620	0.0583	0.0493	0.0254	0.0731	0.0446	0.0463	0.0739				
**SD**	0.0518	0.0675	0.0685	0.0766	0.0718	0.0449	0.0886	0.0615	0.0630	0.0756	0.0564			
**UT**	0.0487	0.0620	0.0641	0.0711	0.0650	0.0349	0.0848	0.0561	0.0589	0.0742	0.0503	0.0625		
**WA**	0.0261	0.0408	0.0458	0.0444	0.0379	0.0144	0.0619	0.0333	0.0335	0.0659	0.0238	0.0433	0.0334	
**CA-SD**	0.0596	0.0755	0.0770	0.0830	0.0766	0.0508	0.0941	0.0665	0.0680	0.0844	0.0624	0.0756	0.0707	0.0494

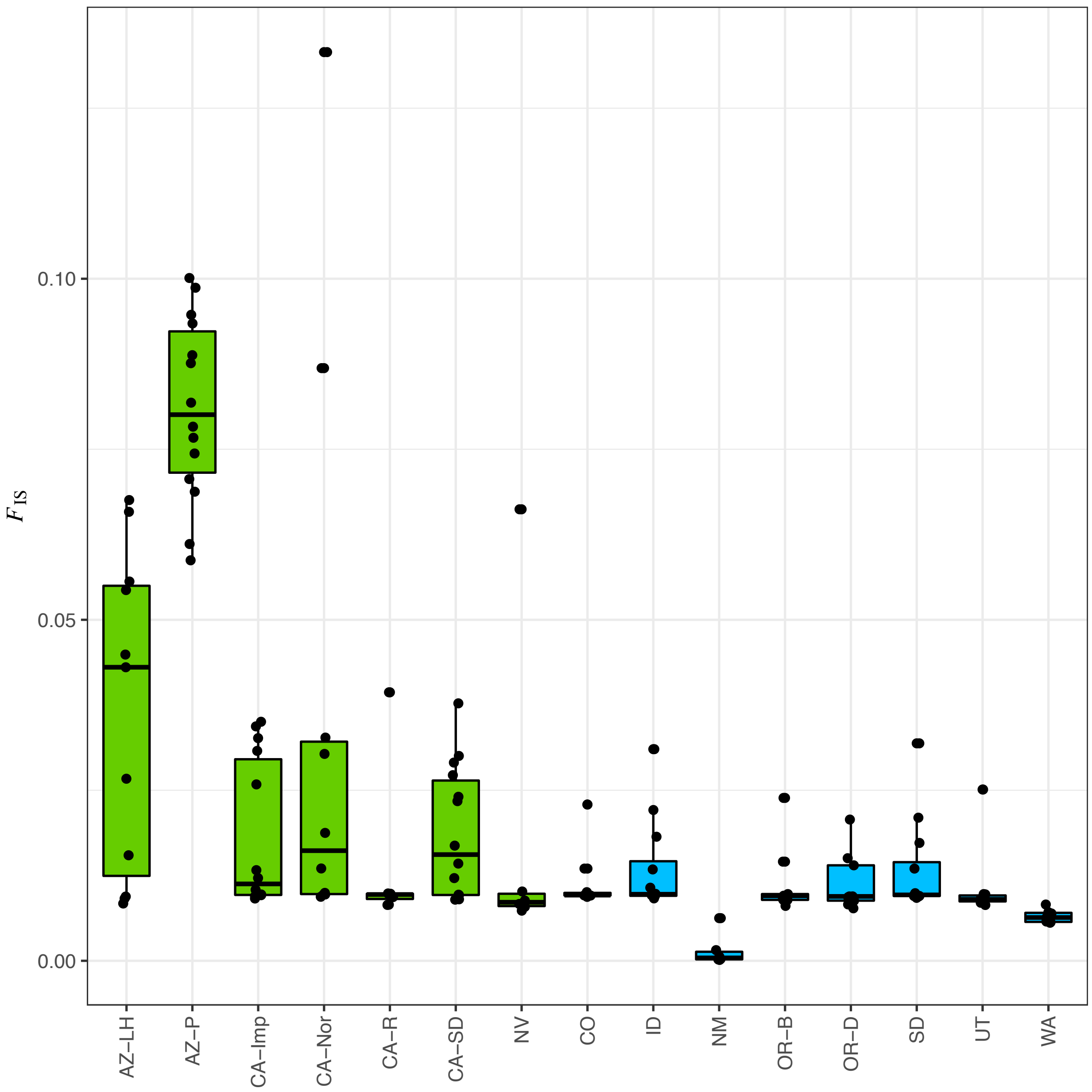

Figure S2. F_{IS} calculated by sample site. Residents are colored green and migrants blue. CA-Nor TO CA-SJ

Table S3. List of genes within 25kbps in *Athene cunicularia cunicularia* annotation using top 0.1% most differentiated loci between *A. c. hypugaea* migrants and residents.

Name	Ensembl Gene ID	Entrez	Gene Type	Species	Chr	Position (Mbp)	Description
CALCR	ENSTGUG00000001514	100223972	protein_coding	Zebra finch	2	24.236071	calcitonin receptor [Source:NCBI gene;Acc:100223972]
NOP14	ENSTGUG00000010455	100220875	protein_coding	Zebra finch	4	9.714675	NOP14 nucleolar protein [Source:NCBI gene;Acc:100220875]
HTT	ENSTGUG00000010399	NA	protein_coding	Zebra finch	4	9.78153	huntingtin [Source:HGNC Symbol;Acc:HGNC:4851]
SORCS2	ENSTGUG00000010129	NA	protein_coding	Zebra finch	4	11.48747	sortilin related VPS10 domain containing receptor 2 [Source:HGNC Symbol;Acc:HGNC:16698]
SPTLC2	ENSTGUG00000012332	NA	protein_coding	Zebra finch	5	40.177954	serine palmitoyltransferase long chain base subunit 2 [Source:HGNC Symbol;Acc:HGNC:11278]
GTF2A1	ENSTGUG00000012378	100219035	protein_coding	Zebra finch	5	41.766624	general transcription factor IIA subunit 1 [Source:NCBI gene;Acc:100219035]
PLCE1	ENSTGUG00000008633	100222852	protein_coding	Zebra finch	6	19.024883	phospholipase C epsilon 1 [Source:NCBI gene;Acc:100222852]
ARMH3	ENSTGUG00000009880	NA	protein_coding	Zebra finch	6	21.589012	armadillo like helical domain containing 3 [Source:HGNC Symbol;Acc:HGNC:25788]
STN1	ENSTGUG00000010354	NA	protein_coding	Zebra finch	6	23.35153	STN1 subunit of CST complex [Source:HGNC Symbol;Acc:HGNC:26200]
ZFYVE9	ENSTGUG00000008916	100231800	protein_coding	Zebra finch	8	7.365492	zinc finger FYVE-type containing 9 [Source:NCBI gene;Acc:100231800]
OSBPL9	ENSTGUG00000008771	100223171	protein_coding	Zebra finch	8	7.542853	oxysterol binding protein like 9 [Source:NCBI gene;Acc:100223171]
CFAP57	ENSTGUG00000007089	100225077	protein_coding	Zebra finch	8	12.666171	cilia and flagella associated protein 57 [Source:NCBI gene;Acc:100225077]
AK5	ENSTGUG00000007008	100229919	protein_coding	Zebra finch	8	13.150598	adenylate kinase 5 [Source:NCBI gene;Acc:100229919]
ZZZ3	ENSTGUG00000007000	100227013	protein_coding	Zebra finch	8	13.250444	zinc finger ZZ-type containing 3 [Source:NCBI gene;Acc:100227013]
TMEM266	ENSTGUG00000003531	100223195	protein_coding	Zebra finch	10	0.781096	transmembrane protein 266 [Source:NCBI gene;Acc:100223195]
ETFA	ENSTGUG00000003549	NA	protein_coding	Zebra finch	10	0.851982	electron transfer flavoprotein subunit alpha [Source:HGNC Symbol;Acc:HGNC:3481]
TLN2	ENSTGUG00000005039	100220667	protein_coding	Zebra finch	10	4.828676	talin 2 [Source:NCBI gene;Acc:100220667]
THSD4	ENSTGUG00000005809	100232194	protein_coding	Zebra finch	10	6.823963	thrombospondin type 1 domain containing 4 [Source:NCBI gene;Acc:100232194]
PEPD	ENSTGUG00000009480	100218638	protein_coding	Zebra finch	11	18.354231	peptidase D [Source:NCBI gene;Acc:100218638]
TRPC7	ENSTGUG00000001207	NA	protein_coding	Zebra finch	13	9.703912	transient receptor potential cation channel subfamily C member 7 [Source:HGNC Symbol;Acc:HGNC:20754]
CLINT1	ENSTGUG00000000611	100226122	protein_coding	Zebra finch	13	13.848802	clathrin interactor 1 [Source:NCBI gene;Acc:100226122]
EBF1	ENSTGUG00000000593	100228980	protein_coding	Zebra finch	13	14.182213	EBF transcription factor 1 [Source:NCBI gene;Acc:100228980]
DUSP1	ENSTGUG00000000299	100219518	protein_coding	Zebra finch	13	16.133668	dual specificity phosphatase 1 [Source:NCBI gene;Acc:100219518]
LOC113481779	Not mapped	NA	NA	NA	NA	NA	NA

Table S4. Gene ontology enrichment analysis using genes within 25kbps of top 0.1% most differentiated loci.

Pathway	Enrichment FDR	Pathway Genes	Fold Enrichment	Genes
Regulation of lipophagy	0.00792242	5	289.026087	HTT, SPTLC2
Positive regulation of lipophagy	0.00792242	5	289.026087	HTT, SPTLC2
Lipophagy	0.011072711	7	206.447205	HTT, SPTLC2

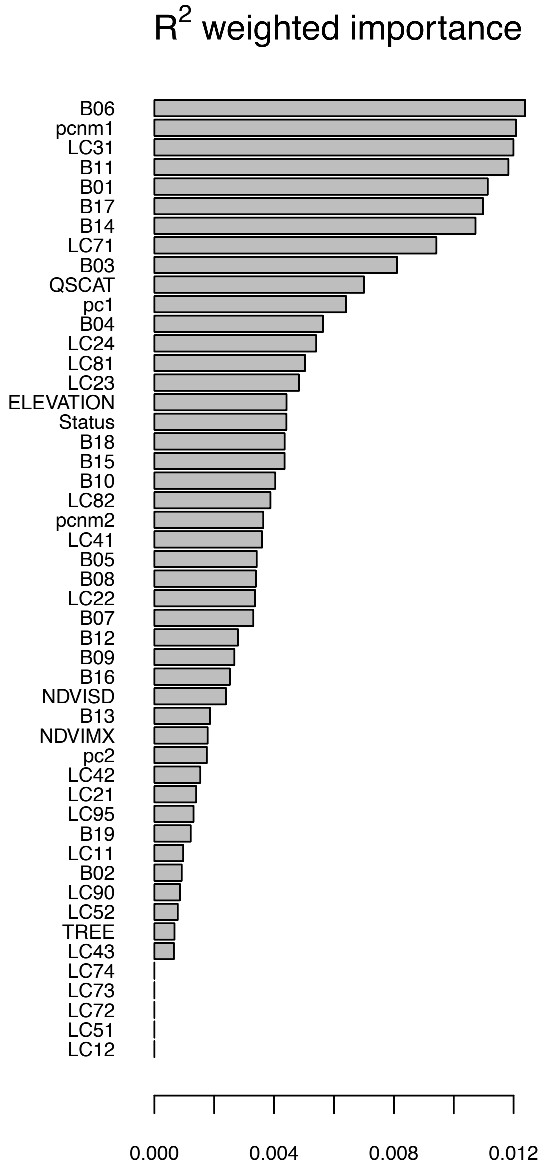

Figure S3. Results of gradient forest analyses using the top 1% of loci most differentiated loci between resident and migrant samples, excluding resident birds sampled at AZ-P and CA-Imp. PCNM1 and B01 are strongly correlated (r>0.75) with B06, hence B06, LC31, B11, and B17 are used for plotting.

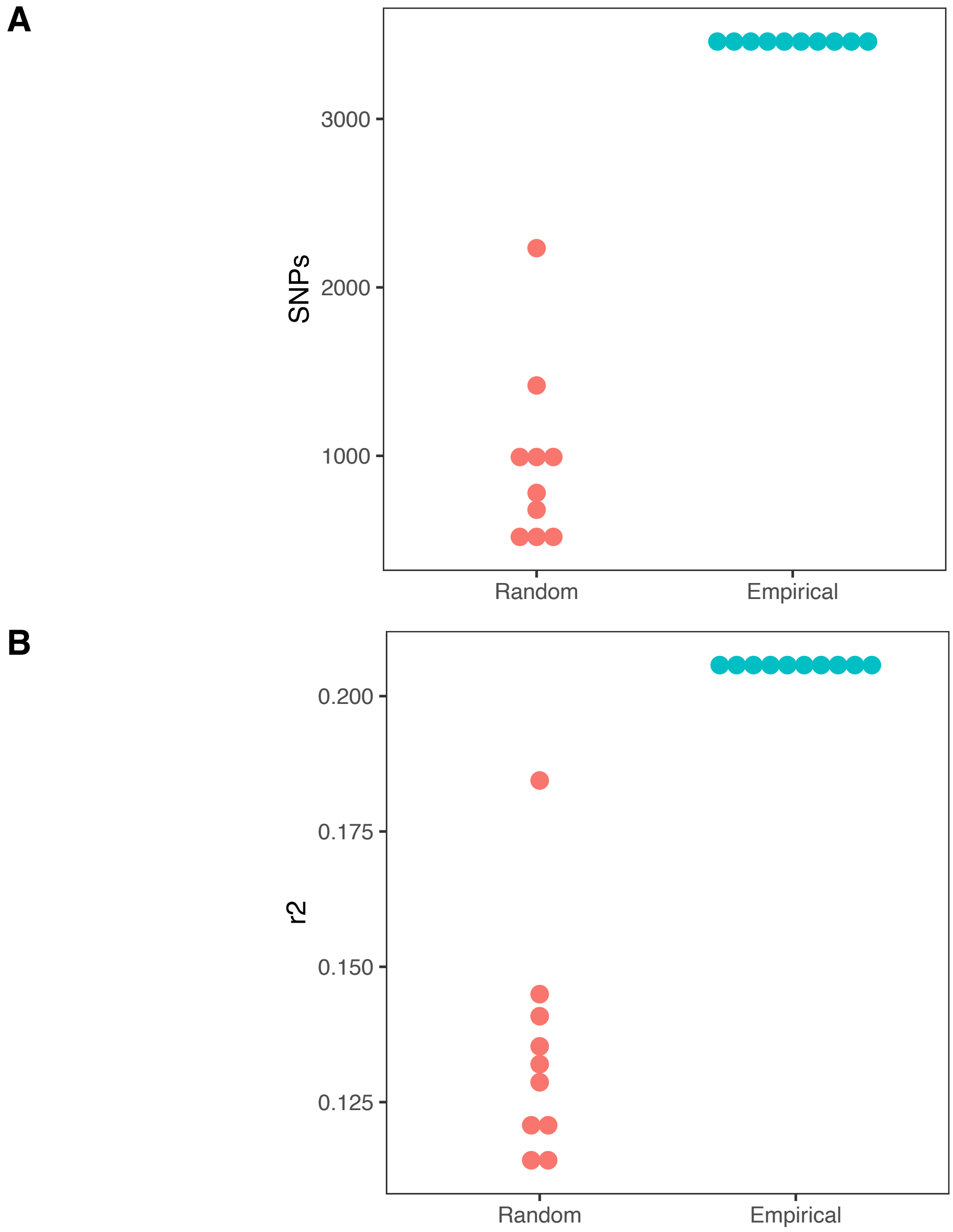

Figure S4. Results of comparisons between total SNPs with positive correlation coefficients (top) and average r2 of those SNPs (bottom) between 10 randomized and 10 empirical gradient forest analyses. Both plots illustrate consistency among empirical analyses, and clearly lower numbers of SNPs and r2s in randomized analyses.

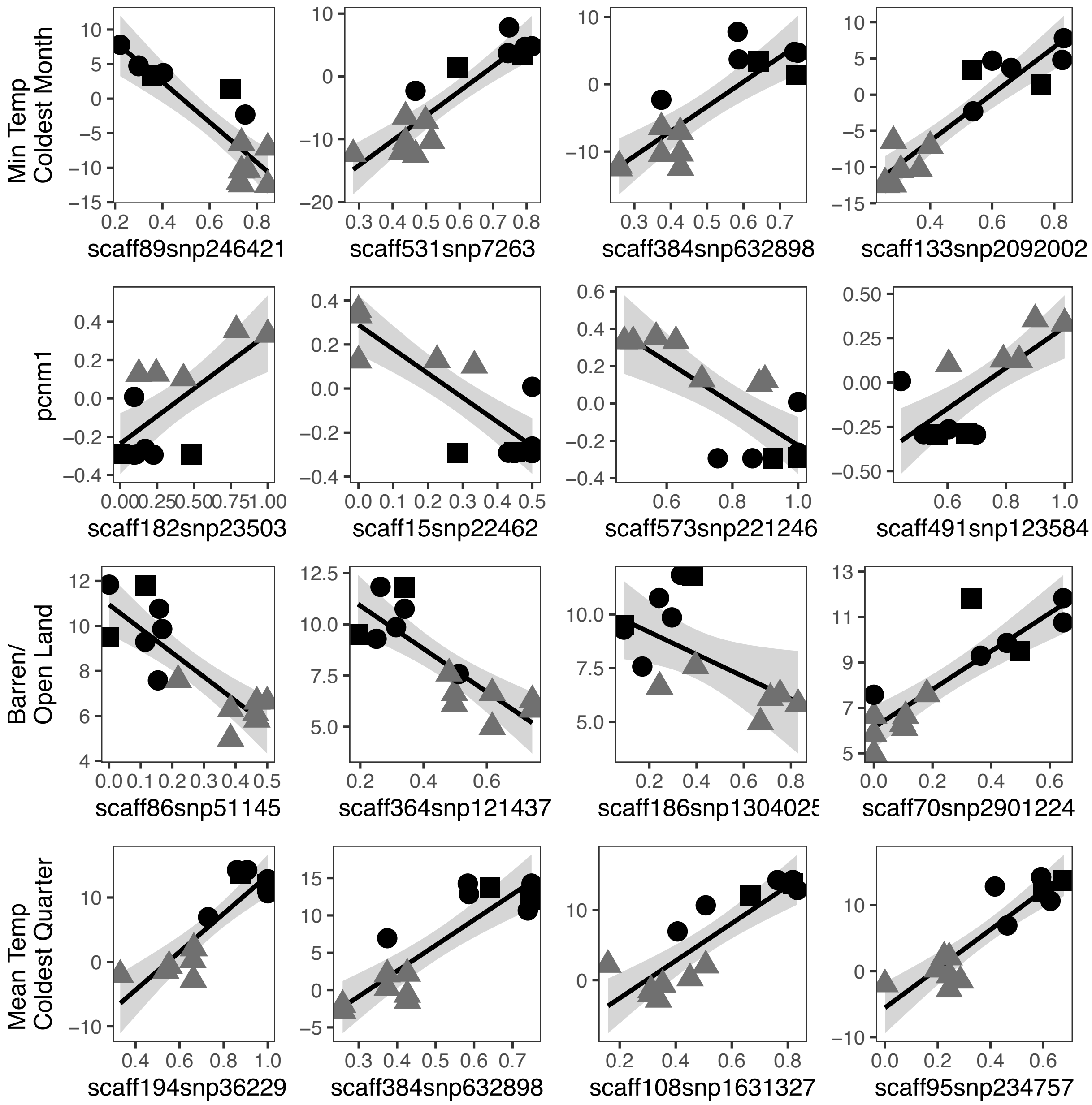

Figure S5. Minor allele frequency trends among resident (circles), migratory (triangles) and switcher (squares) breeding sites at the top four uncorrelated environmental variables identified in gradient forest analyses.

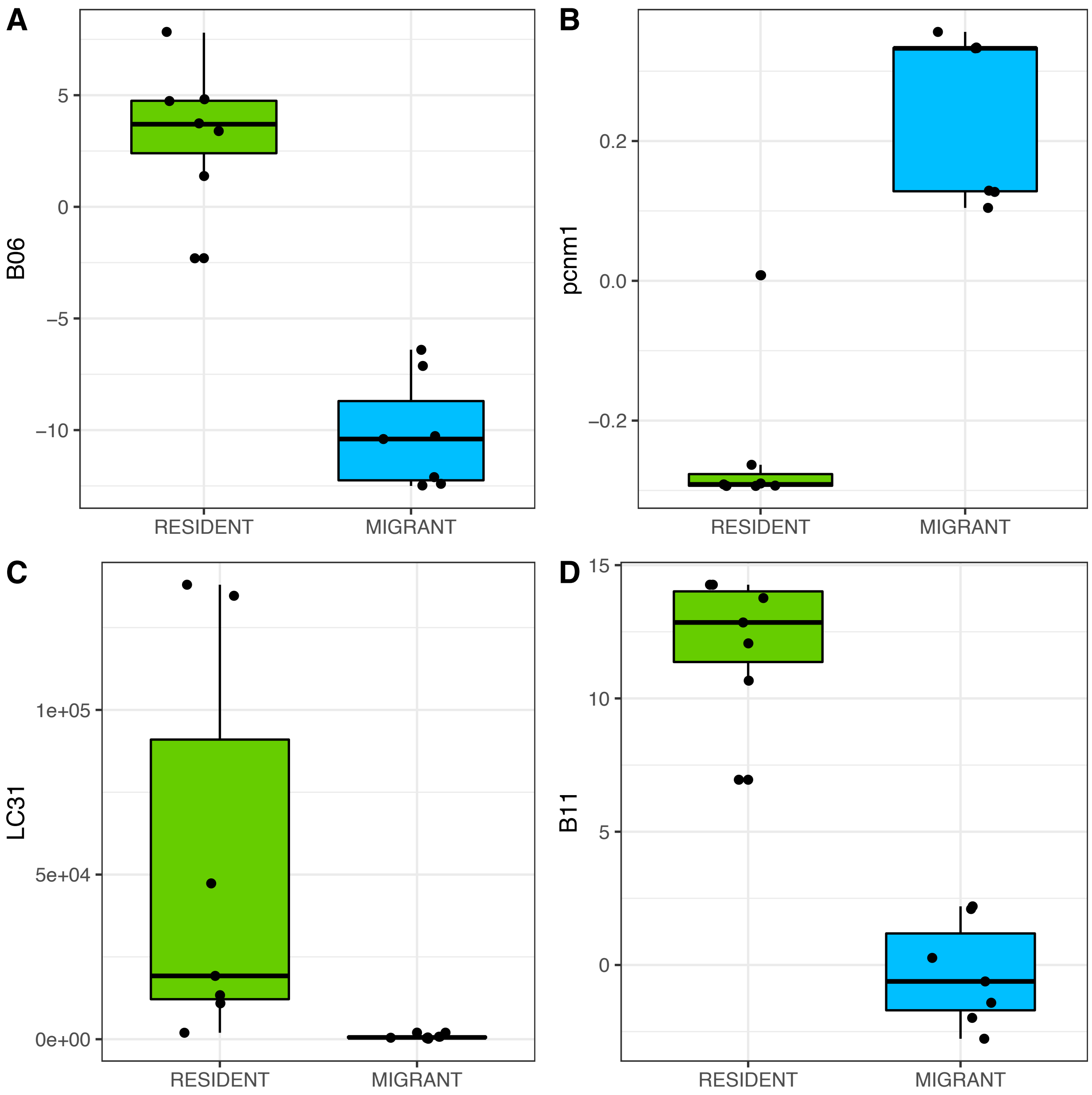

Figure S6. Summaries of top four environmental variables by site type. These do not include New Mexico as the site was excluded from gradient forest analyses. B06 and B11 are bioclimate variables representing, respectively, minimum temperature during the coldest month and mean temperature during the coldest quarter. LC31 is barren/open land as calculated within the National Landcover Dataset. PCNM1 is the first PC from a principal component analysis of neighborhood matrix.
