# Supplementary material for: Genetic and environmental drivers of migratory behavior in western burrowing owls and implications for conservation and management
Source: Evol Appl. 2023 Nov 15;16(12):1889–900. doi: 10.1111/eva.13600 (PMC10739168; doi:10.1111/eva.13600)
Supplement: Supplementary file 1 — Data S1. [file EVA-16-1889-s001.zip › BUOW-supplemental-revision.docx]

Supplemental Methods

*Sample Collection*

Tissue collection was made possible with the assistance of many collaborators (Table S1). We targeted seven resident sites and eight migratory breeding sites. Most sampling entailed capturing individuals in specialized traps (including bownet and box traps), and collecting 10 – 50 uL of blood with a non-heparinized capillary tube following a puncture of the brachial vein (Sheldon et al., 2008; Stangel, 1986) with a sterile 26G X 0.5 mm needle. Collected blood was stored either in Queen’s lysis buffer, in EDTA, on filter paper treated with EDTA, or on Whatman blood cards. Two retrices (i.e., tailfeathers) or five breast feathers were also taken from most adults and these were added to the UCLA Center for Tropical Research’s feather collection. Where possible (e.g., artificial burrows), we obtained growing feathers from nestlings and stored these in Queen’s lysis buffer. We extracted DNA from tissues or snippets of Whatman blood cards using DNeasy extraction kits (Qiagen) following standard procedures, but with the addition of 20 uL of dithiothreitol (DTT) during tissue digestion for growing feathers. We quantified DNA concentrations using a Qubit (Thermofisher) and assessed quality on a 2% agarose gel.

*Genome Assembly and Low Coverage Whole Genome Sequencing*

Our genetic data includes a *de novo* reference genome for *A. c. hypugaea* and low coverage whole genome resequencing. For the former, we prepared DNA for whole genome sequencing using the Illumina TruSeq DNA PCR-Free LT kit (Illumina). After fragmenting 1 µg of DNA to 400 bp using a Diagenode sonicator and cleaning with magnetic beads at a ratio of 105 µL of beads/79 µL of water to select for >400 bp fragments, bioanalyzer traces were collected by the University of California, Los Angeles GenoSeq Core to verify library quality. We sequenced a final library with fragments averaging ~500 bp using a 250 bp paired-end run on an Illumina HiSeq2500 at the University of California QB3 Vincent J. Coates Genomics Sequencing Laboratory. Additional sequence data was also collected using mate-pair libraries (Illumina). Two libraries with insert sizes 4kb and 8kb were prepared at the University of Utah Huntsman Cancer Center and 100 bp paired-end sequencing was run on one-third of an Illumina HiSeq 2500 lane. These data were trimmed and separated from reads sharing the same lane using NxTrim (O’Connell et al., 2015), and SSPACE (Boetzer et al., 2011) was then used to assemble the final scaffolds. Scaffolds were assembled from resulting sequence data via the Discovar DeNovo assembler (Broad Institute), and those <5,000 bp were removed because this improved N50 scores with little cost for assembly completeness. We used BUSCO (Simão et al., 2015) to estimate genome completeness by searching for single copy orthologs common to all species in the class Aves.

We also sequenced 202 samples to low coverage, targeting 1X. For these libraries, DNA concentrations were diluted to ≤ 2.5 ng/uL and prepared for sequencing using Nextera DNA Sample Preparation and Index Kits. DNA was fragmented and tagged with sequencing adaptors in a single step, and then a KAPA Library Amplification Kit (Roche) was used to attach indices. Libraries were cleaned using AMPure beads and 100 bp paired-end reads were sequenced on three lanes of on an Illumina HiSeq2500 at the University of California QB3 Vincent J. Coates Genomics Sequencing Laboratory. We removed duplicates using FastUniq (Xu et al., 2012) and used Trimmomatic (Bolger et al., 2014) to remove adaptors and low quality fragments (SLIDINGWINDOW: 4:15; MINLEN: 36). We then used FLASH (Magoč & Salzberg, 2011) to collapse overlapping reads into single reads and HISAT2 (Kim et al., 2019) to align these to the genome. The clipOverlap module in bamUtil (Jun et al., 2015) was used to clip overlapping pairs, and BEDtools (Quinlan & Hall, 2010) was used for calculating coverage site-by-site for each individual.

Table S1.  Collaborators who either provided samples or provided access to sites for sampling (“collected with assistance”).  Some collaborators provided samples that are not included in this study.  *Specific location is the centerpoint GPS for the general sample sites, and these were used for genotype-environmental analyses.

Figure S1. NGS-Admix results at Ks between 2 - 8, as indicated on the right. Migratory sites were reduced to 3 samples each to facilitate analyses.

Table S2.  Pairwise *F*_ST_ between all sites sampled as calculated using the ‘realSFS’ module in ANGSD.

Figure S2. *F*_IS_ calculated by sample site. Residents are colored green and migrants blue. CA-Nor TO CA-SJ

Table S3.  List of genes within 25kbps in *Athene cunicularia cunicularia* annotation using top 0.1% most differentiated loci between *A. c. hypugaea* migrants and residents.

Table S4.  Gene ontology enrichment analysis using genes within 25kbps of top 0.1% most differentiated loci.

Figure S3. Results of gradient forest analyses using the top 1% of loci most differentiated loci between resident and migrant samples, excluding resident birds sampled at AZ-P and CA-Imp. PCNM1 and B01 are strongly correlated (r>0.75) with B06, hence B06, LC31, B11, and B17 are used for plotting.

Environmental Data:

BIO1: Annual Mean Temperature

BIO2: Mean Diurnal Range

BIO3: Isothermality

BIO4: Temperature Seasonality

BIO5: Max Temperature of the Warmest Month

BIO6: Min Temperature of the Warmest Month

BIO7: Temperature Annual Range

BIO8: Mean Temperature of the Wettest Quarter

BIO9: Mean Temperature of the Driest Quarter

BIO10: Mean Temperature of the Warmest Quarter

BIO11: Mean Temperature of the Coldest Quarter

BIO12: Annual Precipitation

BIO13: Precipitation of the Wettest Month

BIO14: Precipitation of the Driest Month

BIO15: Precipitation Seasonality

BIO16: Precipitation of the Wettest Quarter

BIO17: Precipitation of the Driest Quarter

BIO18: Precipitation of the Warmest Quarter

BIO19: Precipitation of the Coldest Quarter

NDVI_Mean: Mean Normalized Difference Vegetation Index

NDVI StDev: Standard Deviation of Normalized Difference Vegetation Index

SRTM: Elevation

QuickSCAT: Surface Moisture

LC11: Open Water; areas of open water, generally with less than 25% cover of vegetation

or soil.

LC12: Perennial Ice/Snow; areas characterized by a perennial cover of ice and/or snow,

generally greater than 25% of total cover.

LC21: Developed, Open Space: areas with a mixture of some constructed materials, but

mostly vegetation in the form of lawn grasses. Impervious surfaces account for less

than 20% of total cover. These areas most commonly include large-lot single-family

housing units, parks, golf courses, and vegetation planted in developed settings for

recreation, erosion control, or aesthetic purposes.

LC22: Developed, Low Intensity; areas with a mixture of constructed materials and

vegetation. Impervious surfaces account for 20% to 49% percent of total cover.

These areas most commonly include single-family housing units.

LC23: Developed, Medium Intensity: areas with a mixture of constructed materials and

vegetation. Impervious surfaces account for 50% to 79% of the total cover. These

areas most commonly include single-family housing units.

LC31: Barren Land (Rock/Sand/Clay); areas of bedrock, desert pavement, scarps, talus,

slides, volcanic material, glacial debris, sand dunes, strip mines, gravel pits and other

accumulations of earthen material. Generally, vegetation accounts for less than 15%

of total cover.

LC41: Deciduous Forest; areas dominated by trees generally greater than 5 meters tall,

and greater than 20% of total vegetation cover. More than 75% of the tree species

shed foliage simultaneously in response to seasonal change.

LC42: Evergreen Forest; areas dominated by trees generally greater than 5 meters tall,

and greater than 20% of total vegetation cover. More than 75% of the tree species

maintain their leaves all year. Canopy is never without green foliage.

LC43: Mixed Forest; areas dominated by trees generally greater than 5 meters tall, and

greater than 20% of total vegetation cover. Neither deciduous nor evergreen species

are greater than 75% of total tree cover.

LC51: Dwarf Scrub; Alaska only areas dominated by shrubs less than 20 centimeters tall

with shrub canopy typically greater than 20% of total vegetation. This type is often

co-associated with grasses, sedges, herbs, and non-vascular vegetation.

LC52: Shrub/Scrub; areas dominated by shrubs; less than 5 meters tall with shrub canopy

typically greater than 20% of total vegetation. This class includes true shrubs, young

trees in an early successional stage or trees stunted from environmental conditions.

LC71: Grassland/Herbaceous; areas dominated by gramanoid or herbaceous vegetation,

generally greater than 80% of total vegetation. These areas are not subject to

intensive management such as tilling, but can be utilized for grazing.

LC72: Sedge/Herbaceous; Alaska only areas dominated by sedges and forbs, generally

greater than 80% of total vegetation. This type can occur with significant other

grasses or other grass like plants, and includes sedge tundra, and sedge tussock

tundra.

LC73: Lichens; Alaska only areas dominated by fruticose or foliose lichens generally

greater than 80% of total vegetation.

LC74: Moss; Alaska only areas dominated by mosses, generally greater than 80% of total

vegetation.

LC81: Pasture/Hay; areas of grasses, legumes, or grass-legume mixtures planted for

livestock grazing or the production of seed or hay crops, typically on a perennial

cycle. Pasture/hay vegetation accounts for greater than 20% of total vegetation.

LC82: Cultivated Crops; areas used for the production of annual crops, such as corn,

soybeans, vegetables, tobacco, and cotton, and also perennial woody crops such as

orchards and vineyards. Crop vegetation accounts for greater than 20% of total

vegetation. This class also includes all land being actively tilled.

LC90: Woody Wetlands; areas where forest or shrubland vegetation accounts for greater

than 20% of vegetative cover and the soil or substrate is periodically saturated with

or covered with water.

LC95: Emergent Herbaceous Wetlands; Areas where perennial herbaceous vegetation

accounts for greater than 80% of vegetative cover and the soil or substrate is

periodically saturated with or covered with water.

Figure S4. Results of comparisons between total SNPs with positive correlation coefficients (top) and average r2 of those SNPs (bottom) between 10 randomized and 10 empirical gradient forest analyses. Both plots illustrate consistency among empirical analyses, and clearly lower numbers of SNPs and r2s in randomized analyses.

Figure S5. Minor allele frequency trends among resident (circles), migratory (triangles) and switcher (squares) breeding sites at the top four uncorrelated environmental variables identified in gradient forest analyses.

Figure S6. Summaries of top four environmental variables by site type.  These do not include New Mexico as the site was excluded from gradient forest analyses. B06 and B11 are bioclimate variables representing, respectively, minimum temperature during the coldest month and mean temperature during the coldest quarter.  LC31 is barren/open land as calculated within the National Landcover Dataset.  PCNM1 is the first PC from a principal component analysis of neighborhood matrix.
